# Iron-Terephthalate Coordination Network Thin Films Through In-Situ Atomic/Molecular Layer Deposition

**DOI:** 10.1038/s41598-018-27124-7

**Published:** 2018-06-12

**Authors:** A. Tanskanen, M. Karppinen

**Affiliations:** Department of Chemistry and Materials Science, Aalto University, P.O. Box 16100, FI-00076 Aalto, Finland

## Abstract

Iron terephthalate coordination network thin films can be fabricated using the state-of-the-art gas-phase atomic/molecular layer deposition (ALD/MLD) technique in a highly controlled manner. Iron is an Earth-abundant and nonhazardous transition metal, and with its rich variety of potential applications an interesting metal constituent for the inorganic-organic coordination network films. Our work underlines the role of the metal precursor used when aiming at *in-situ* ALD/MLD growth of crystalline inorganic-organic thin films. We obtain crystalline iron terephthalate films when FeCl_3_ is employed as the iron source whereas depositions based on the bulkier Fe(acac)_3_ precursor yield amorphous films. The chemical composition and structure of the films are investigated with GIXRD, XRR, FTIR and XPS.

## Introduction

Crystalline inorganic-organic hybrids or so-called coordination network structures are metal complexes formed by metal cation centers and organic ligand linkers and extended to one, two or three dimensions^1,2^. The variety of different coordination network compounds is huge, and they are currently intensely investigated owing to their exciting property palette relevant to many frontier research fields and future applications. The wide property palette derives from their (i) hybrid chemical compositions involving both inorganic and organic moieties, (ii) readily engineered crystal structures with e.g. tunable porosities, and (iii) possible guest species accommodated in the pores.

From the potential applications point of view, it would be advantageous to be able to produce the coordination network material in thin-film form^3,4^. While the synthesis techniques for these materials in bulk form are well established, the availability of appropriate thin-film deposition techniques is much more limited. In particular, the direct gas-phase deposition of these materials using the atomic/molecular layer deposition (ALD/MLD) technique was reported only very recently^5–7^. The ALD/MLD technique is an elegant way to link inorganic and organic entities together through gas-surface reactions of sequentially pulsed gaseous precursors^8–11^. It is an extension of the state-of-the-art ALD (atomic layer deposition) technique of inorganic materials, extensively used e.g. in microelectronics^12^. For the inorganic-organic thin films, ALD precursor pulses are combined with MLD (molecular layer deposition) pulses of an organic precursor. This enables the growth of hybrid inorganic-organic thin films with atomic/molecular level precision for the film thickness and composition in a manner parallel to the growth of purely inorganic thin films through conventional ALD.

Even though ALD/MLD is in principle uniquely suited to deposit high-quality thin films of the coordination network materials, the inorganic-organic films enabled by ALD/MLD are not necessarily crystalline. So far *in-situ* crystalline films have been realized by ALD/MLD mostly for the most electropositive cation constituents only, that is, for the s-block metals and lanthanum^5,6,13–16^. As these cations tend to form ionic bonds with organic linkers, it thus seems that the non-directionality of ionic bonding could promote the *in-situ* crystallization during the ALD/MLD process by providing the organic molecules with more freedom for the proper orientation.

Transition metals with partly filled d orbitals and less electropositive nature prefer directional covalent bonds. Most of the ALD/MLD processes for transition metals have yielded amorphous films^17–21^, the only exceptions being the ALD/MLD processes for copper^7^ and zirconium^22,23^ based on Cu(thd)_2_ (thd: 2,2,6,6-tetramethyl-3,5-heptanedione) plus TPA (TPA: terephthalic acid or 1,4-benzene dicarboxylic acid), ZrCl_4_ plus TPA and ZrCl_4_ plus 2-amino derivative of TPA. Note, however, that in the latter case an additional post-deposition treatment was required to crystallize the films. Also, tetravalent zirconium does not possess partly filled d orbitals.

Here we report for the first time an ALD/MLD process for crystalline iron terephthalate. Iron is an abundant and environmentally benign transition metal element, exhibiting e.g. interesting electrical and magnetic properties in its compounds. A number of Fe-TP coordination network structures are known in bulk form, i.e. (Fe(OH(TP) ∙ (solv)x (MIL-53)^24^, (Fe_2_O(O_2_CCH_3_)_2_(TP) ∙ (solv)_x_ (MIL-85)^25^, (Fe_3_O(OH)(TP)_3_ ∙ (solv)_x_ (MIL-88B/MOF 235)^26,27^ and (Fe(OH)(TP) ∙ (dmf)_x_ (MIL-68)^24^; moreover, these materials are currently investigated for applications such as Li-ion battery^24,25,28^, catalysis^26,27^ and drug delivery^28^. For the ALD/MLD growth of crystalline iron terephthalate thin films, the choice of the iron precursor turned out to be crucial, as crystalline films were obtained from FeCl_3_, while the use of Fe(acac)_3_ (acac: acetylacetonate) as the iron source resulted in amorphous films. We tentatively attribute this difference to the different sizes of the ligands in these two iron precursors.

## Results and Discussion

### Crystallinity

**Figure 1:**
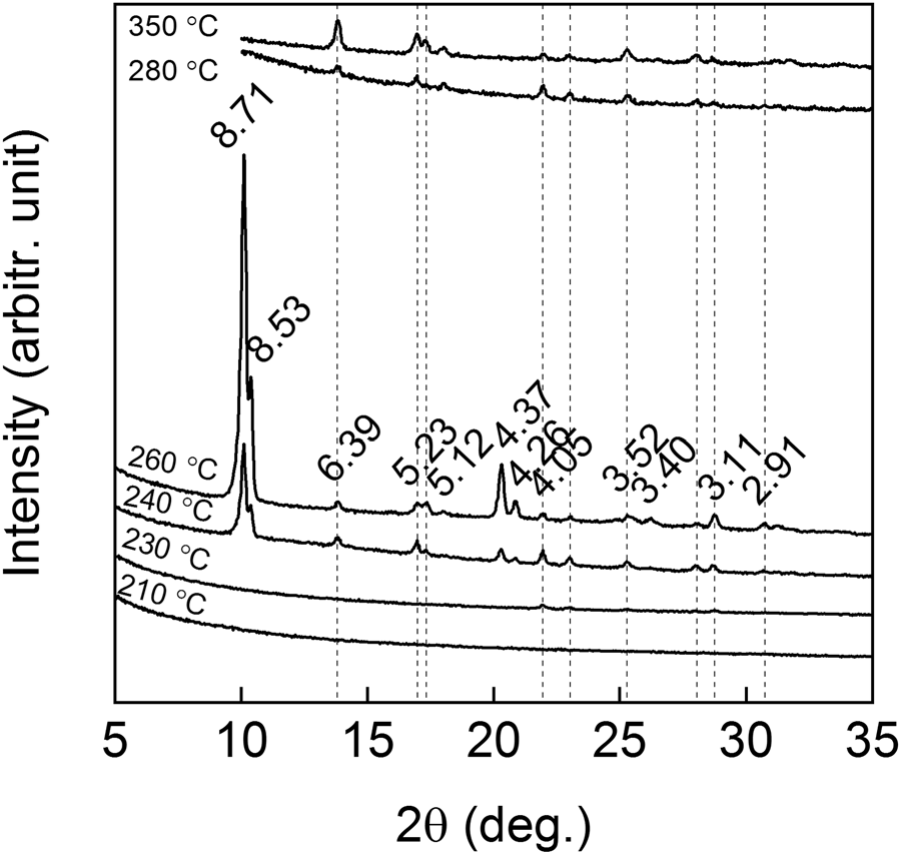
GIXRD patterns for Fe-TP films deposited through the FeCl_3_+ TPA process at different temperatures; the diffraction peaks are indicated with their d values.

We initially selected two common ALD iron precursors, FeCl_3_ and Fe(acac)_3_, to be combined with our organic precursor of choice, i.e. TPA, and mapped the deposition temperature ranges where crystalline films were obtained; in these experiments the lowest temperatures (defined by the evaporation temperatures of the precursors) were 200 °C for the FeCl_3_+ TPA process and 230 °C for the Fe(acac)_3_+ TPA process. For the FeCl_3_+ TPA process a relatively wide deposition temperature range was found within which the films were crystalline; on the other hand, all the films from the Fe(acac)_3_+ TPA process independent of the deposition temperature turned out to be amorphous. Thus, in Fig. 1 we show representative grazing incidence X-ray diffraction (GIXRD) patterns only for the FeCl_3_+ TPA process. It is seen that the films deposited at 230 °C or below are essentially amorphous, but for the films deposited at 240 °C or at higher temperatures clear diffraction peaks appear with increasing intensity up to 260 °C. An important observation concerned the film roughness values which we estimated for the films from the X-ray reflectivity (XRR) patterns (not shown). It was seen that the roughness – as expected – clearly increased for the crystalline films. However, interestingly the roughness value (being less than 2 nm for a ca. 50-nm thick film deposited at 210 °C) started to increase already at 220 °C (to ca. 3 nm), suggesting that already the films deposited at 210 °C could be partly crystalline. Then, for the films deposited at 280 °C and higher temperatures the most intense diffraction peaks around 10° and 20° disappear, while some other peaks rather gain intensity slightly, presumably due to changes in phase composition, crystal structure or orientation.

Comparison of the GIXRD patterns to the crystal structure data/diffraction patterns reported in literature^24,26,29–34^ for different iron terephthalate structures did not yield any perfect match. This is understandable though, as the wet-chemically synthesized bulk samples – differently from our gas-phase deposited thin films – are likely to contain some solvent molecules in the structure. Actually, there are several examples of crystalline structures discovered for ALD/MLD grown inorganic-organic thin films^13–16^ without previous reports of similar crystalline phases in bulk samples synthesized through conventional solution techniques. The closest resemblance was seen to the so-called MOF-2 structure reported for Cu-TP and Zn-TP^29,35^. This structure was also concluded for our ALD/MLD grown Cu-TP films^5^. Here however the experimental GIXRD pattern is not fully explained by the MOF-2 structure, the most visible difference being the low-angle double reflection seen for our Fe-TP thin films instead of the single peak expected for the MOF-2 structure.

### Chemical state and bonding structure

**Figure 2:**
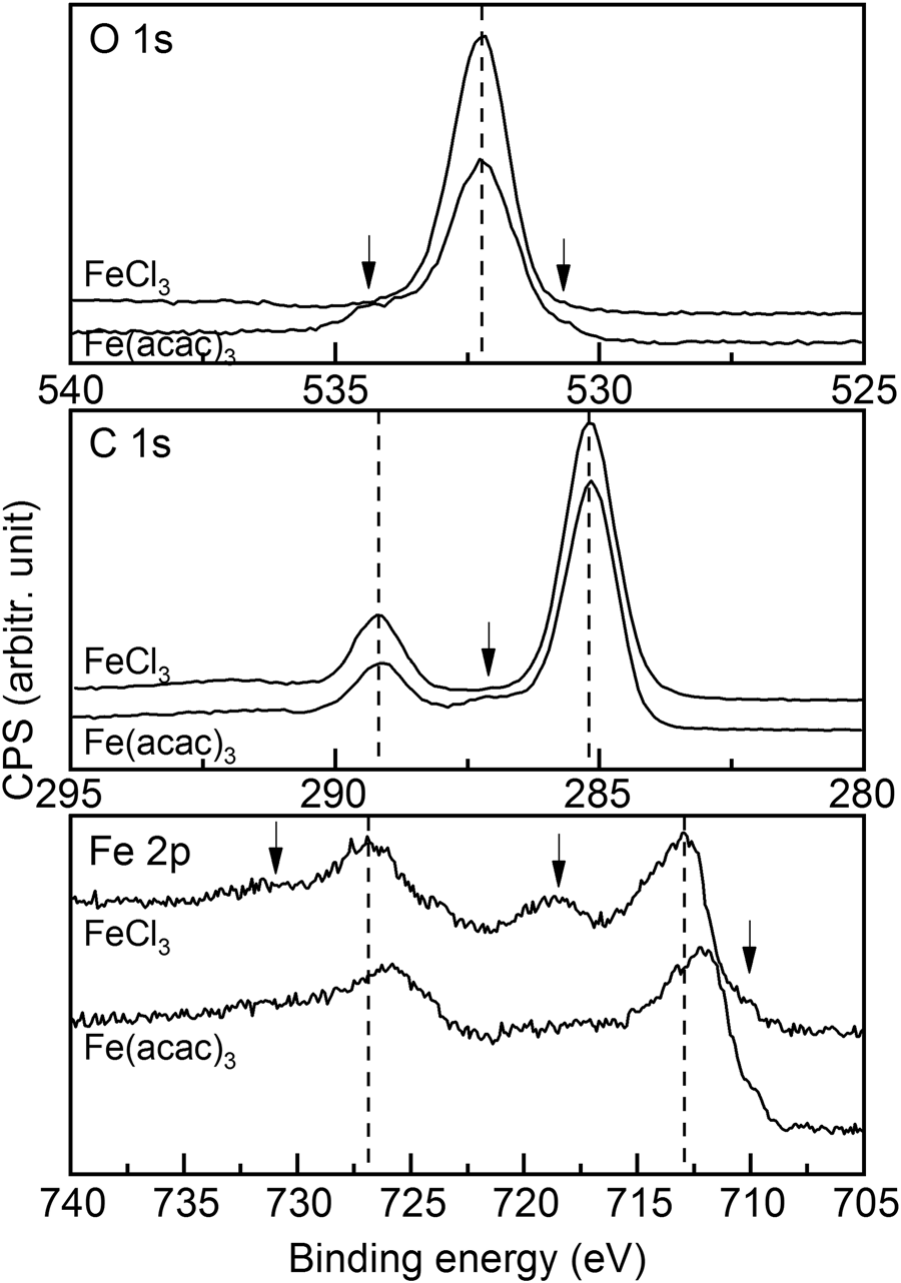
High-resolution XPS spectra for a crystalline Fe-TP film deposited through the FeCl_3_+ TPA process and an amorphous Fe-TP film deposited through the Fe(acac)_3_+ TPA process, in both cases at 250 °C.

Chemical composition and the iron valence state in the films from the two processes was studied using X-ray photoelectron spectroscopy (XPS). Firstly, the wide-scan spectra (not shown) confirmed that none of the films contained detectable amounts of elements other than iron, carbon and oxygen. This is an important notion for the films deposited through the FeCl_3_+ TPA process, as they could contain chlorine as an unintentional impurity. The high-resolution Fe 2p, C 1 s and O 1 s spectra are shown in Fig. 2 for two samples deposited at 250 °C, one from the FeCl_3_+ TPA process and another from the Fe(acac)_3_+ TPA process. The main O 1 s peak seen for both samples around 532 eV is compatible with oxygen in the C-COOH environment^36^, while in the C 1 s spectra, the main peaks around 285 and 290 eV are due to C-C and alpha-carbon in the carboxylic group, respectively^37^. For the film grown from Fe(acac)_3_ additional shoulder peaks are seen in both the O 1 s and C 1 s spectra, which could originate from the larger variety in bonding in the amorphous film.

For both the samples, the Fe 2p spectra show the Fe 2p_1/2_ and 2p_3/2_ peaks, but they appear at lower energies for the amorphous film grown from Fe(acac)_3_, indicating towards the lower oxidation state of iron. On the other hand, for the crystalline film grown from FeCl_3_ a clear satellite is observed between the main peaks; this is typical for hematite with trivalent iron but not for magnetite with mixed-valence iron^38^. The two additional satellites around 732 and 710 eV seen for this film are most likely due to iron bonded to carboxylic acid^37^. Thus, to summarize our findings from the XPS data, it seems that iron is trivalent in our crystalline Fe-TP film obtained from the FeCl_3_+ TPA process but possibly slightly reduced in the amorphous film obtained from the Fe(acac)_3_+ TPA process.

**Figure 3:**
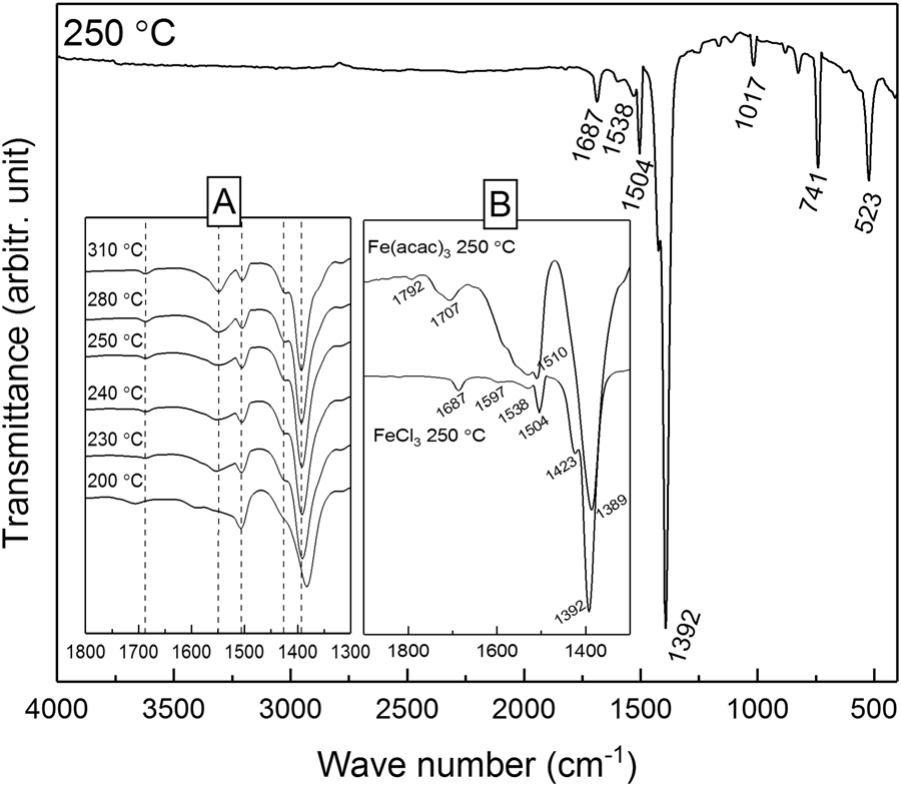
FTIR spectra for Fe-TP films deposited through the FeCl_3_+ TPA process at 250 °C, and in inset A for films deposited at different temperatures. Inset B compares the spectra for a crystalline Fe-TP film from the FeCl_3_+ TPA process and an amorphous Fe-TP film from the Fe(acac)_3_+ TPA process.

**Figure 4:**
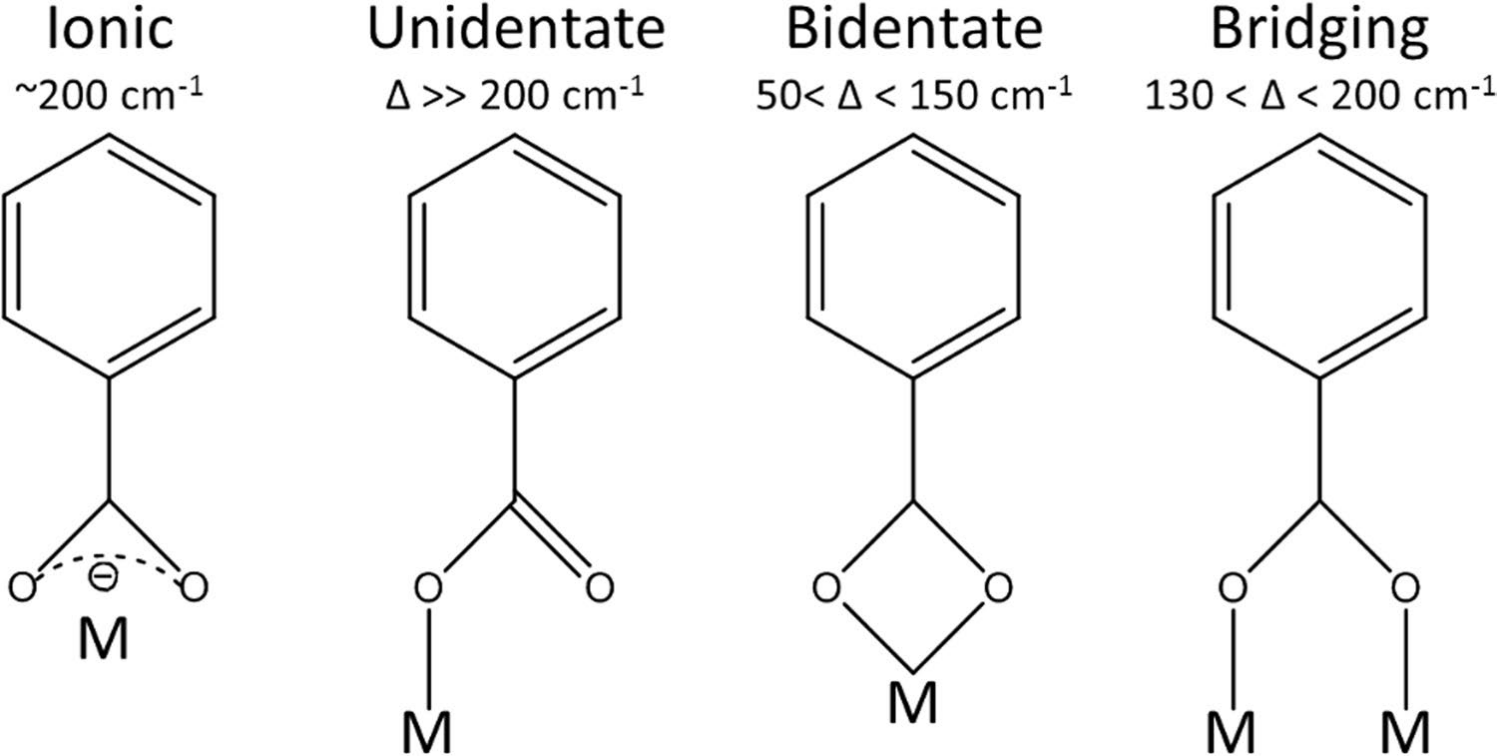
Possible binding types of carboxylate anion to the metal cation and the corresponding peak separation distances (Δ) of the asymmetric and symmetric vibrations of the carboxylic group^41,42^.

In Fig. 3 we show Fourier transform infrared (FTIR) spectra for a series of Fe-TP films deposited through the the FeCl_3_+ TPA process at different temperatures. In overall, the spectra are very similar to those reported for terephthalate-based coordination framework bulk materials^39,40^; the main absorption bands for the carboxylate group are seen at 1504 and 1392 cm^−1^, and the characteristic C-H vibration of the benzene ring at 741 cm^−1^. In the inset A of Fig. 3, the peak positions in the 1400–1700 cm^−1^ area are examined with the deposition temperature. In particular, the separation (Δ) between the symmetric (ν_a_) and asymmetric (ν_as_) carboxylic group vibration bands indicates the type of the metal-carboxylate bonding, i.e. ionic (Δ ≫ 200 cm^−1^), unidentate (Δ» 200 cm^−1^), bidentate (50 < Δ < 150 cm^−1^) or bridging (130 < Δ < 200 cm^−1^) type, see Fig. 4^41,42^. Most importantly, for the crystalline film deposited at 250 °C, the Δ value is 112 cm^−1^ thus clearly indicating towards the bidentate bonding type. With decreasing deposition temperature, Δ slightly increases, but even the value of 125 cm^−1^ observed for the amorphous film deposited at 200 °C is still typical for the bidentate type. In the inset B of Fig. 3, we compare the FTIR spectra for a crystalline Fe-TP film from the FeCl_3_+ TPA process and an amorphous Fe-TP film from the Fe(acac)_3_+ TPA process, both deposited at 250 °C. For the latter amorphous film the peaks are much broader, indicating a larger variety in the types of bonding compared to the more uniform bonding in the crystalline films.

**Figure 5:**
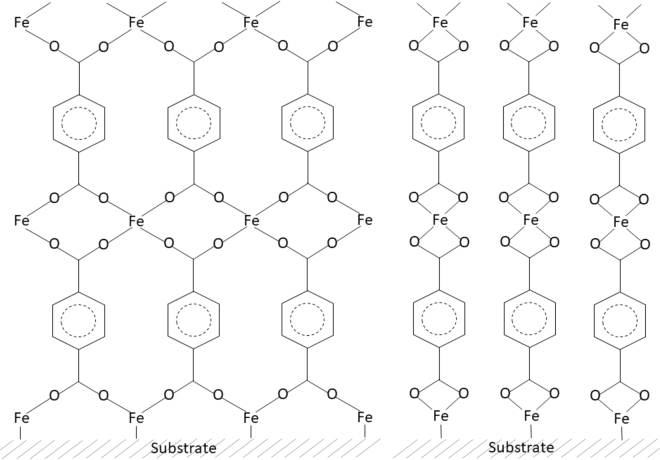
Schematic 2D illustrations of the two possible structures/film growth mechanisms with bridging (left) and bidentate (right) types of bonding.

Based on the FTIR, XPS and GIXRD results discussed above we schematically illustrate in Fig. 5 two possible 2D models for the growth mechanism/bonding structure in our Fe-TP thin films. It is known in general that for metal terephthalates based on trivalent (or higher valence) metal species with higher coordination numbers both the bidentate and bridging bonding types between the carboxylate anion and the metal cation are common, and preferred over the other types of bonding^43^. In Fig. 5, the structure on the left based on bridging type carboxylates is in accordance with the MOF-2 crystal structure reported for some terephthalates of divalent metal species, i.e. Cu^2+^ and Zn^2+^ ^29,35^. However, our GIXRD patterns were not perfectly compatible with the MOF-2 structure, although being quite similar. Then, on the right we show a structure which is consistent with our FTIR data in particular for the crystalline films clearly indicating towards bidentate carboxylate groups. Finally, it should be recalled that for the amorphous films the FTIR features were broader and pointed towards a possible coexistence of both bridging and bidentate bonding schemes.

### ALD/MLD growth characteristics

All the depositions yielded visually homogeneous thin films. We determined the film thicknesses from the XRR data to calculate the growth-per-cycle (GPC) values, taken as the film thickness divided by the number of ALD/MLD cycles applied. For the crystalline films deposited in the temperature range of 240–260 °C, a GPC value of ca. 11 Å/cycle was obtained. This is roughly on the level one would expect on the bases of the length of one Fe-TP unit. In the amorphous area at the deposition temperature of 230 °C and below higher GPC values up to ca. 20 Å/cycle were observed the reason of which is not fully understood. For the films deposited above 280 °C the strongly increased roughness prevented the thickness determination by XRR.

**Figure 6:**
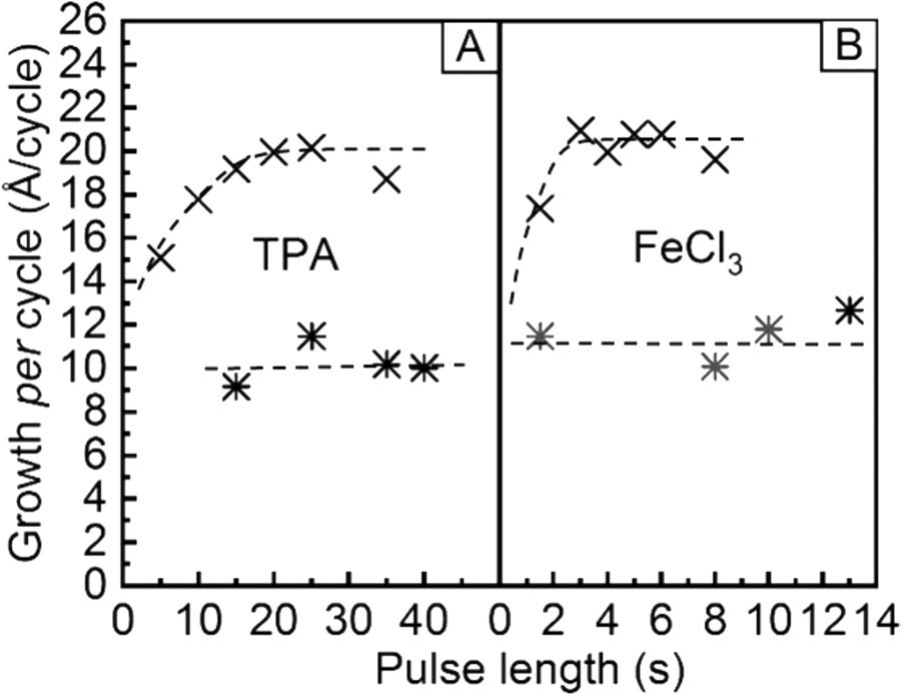
Saturation of the GPC values with increasing precursor pulse lengths of (A) TPA and (B) FeCl_3_ at the deposition temperatures of 210 °C (x) and 250 °C (*). Dashed lines are guides to the eye.

To verify that our FeCl_3_+ TPA process essentially follows the layer-by-layer growth expected for an ideal ALD/MLD process, we investigated the saturation of the surface reactions by following the saturation of the GPC value with increasing precursor pulse lengths, as shown in Fig. 6. These experiments were carried out at both 210 and 250 °C. It can be seen from Fig. 6 that at 210 °C, saturation is reached with the pulse/N_2_ purge times of 4 s/8 s for FeCl_3_ and 25 s/50 s for TPA, while at 250 °C the saturation could be achieved even with somewhat shorter pulse lengths. It should be noted that in most of the depositions discussed earlier we had fixed the precursor pulse lengths to 4 s for FeCl_3_ and 20 s for TPA.

**Figure 7:**
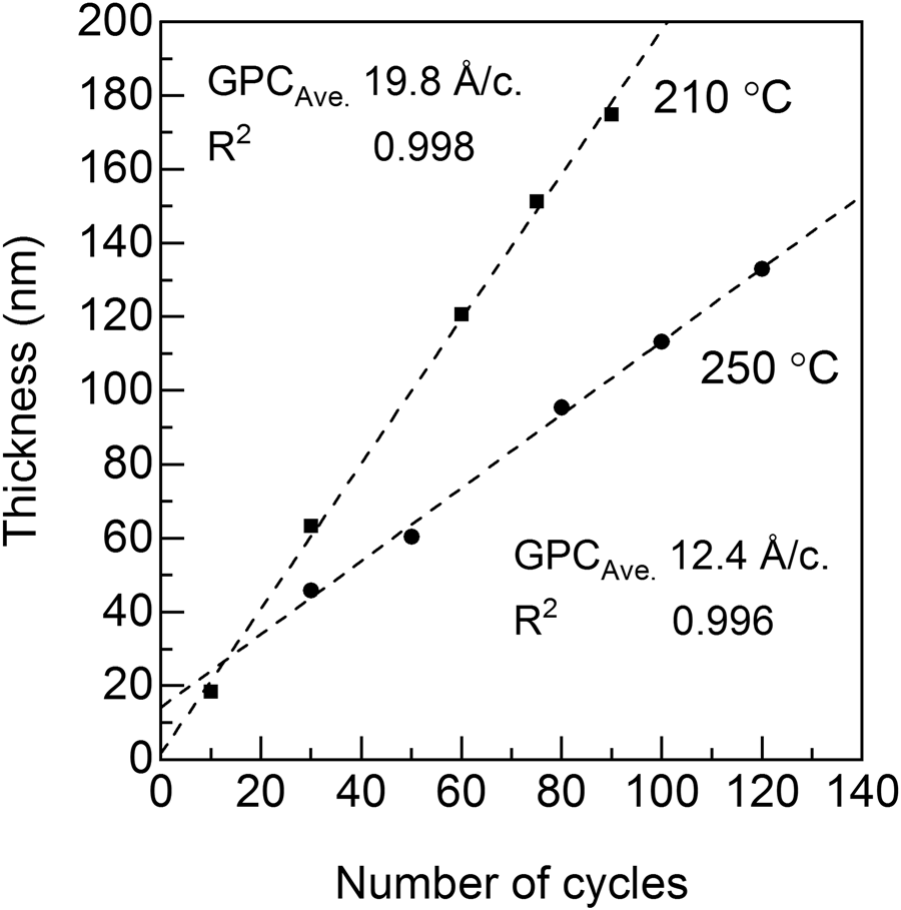
Film thickness versus number of ALD/MLD cycles applied for Fe-TP films deposited through the FeCl_3_+ TPA process at 210 and 250 °C.

Finally, we confirmed that the film thickness in our FeCl_3_+ TPA process is precisely controlled by the number of ALD/MLD cycles applied, see Fig. 7. This is another criterion of an ALD/MLD type growth, and also one of the key issues when considering the applicability of a thin film process. From Fig. 7, the film thickness indeed linearly increases with the number of deposition cycles at the two representative deposition temperatures selected, i.e. 210 °C for the amorphous films and 250 °C for the crystalline films. The fact that the extrapolations of the data points end up to slightly different values at the point of zero cycles can be attributed to different substrate effects on the growth of the amorphous and crystalline materials, or the dissimilar nature in nucleation of the growth at different deposition temperatures^44^.

### Stability of the films

**Figure 8:**
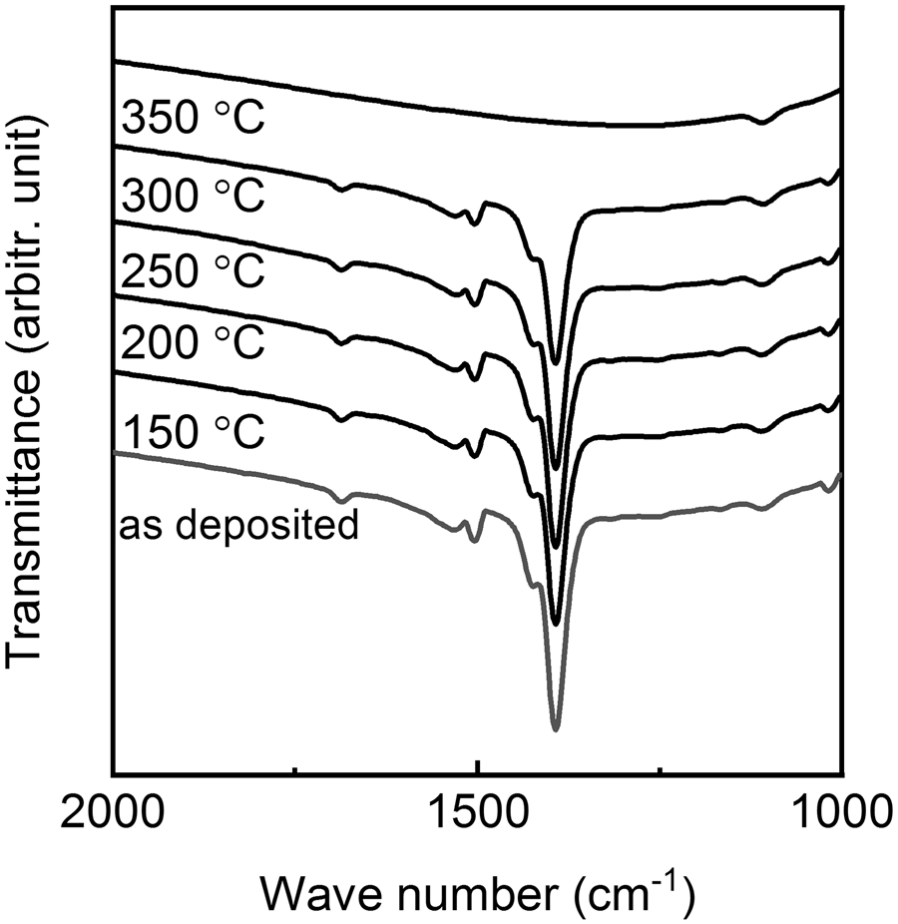
FTIR spectra recorded for a Fe-TP film (deposited through the FeCl_3_+ TPA process at 280 °C) after heat treating it in air at different temperatures up to 350 °C.

All the films were found stable in ambient conditions; we investigated several films after a storage of several weeks and no changes in GIXRD or XRR patterns or FTIR spectra were seen. Moreover, the thermal stability of the films was investigated by annealing a representative crystalline film (deposited at 280 °C) in air at different temperatures; after each heat treatment the film was then examined by GIXRD, XRR and FTIR. From Fig. 8 where we show the FTIR spectra it can be seen that the film remains unaffected up to 300 °C above which all the features in the FTIR spectrum disappear. Similarly, both the GIXRD and XRR data confirmed the stability of the film up to 300 °C.

## Conclusion

We have developed a facile ALD/MLD process to deposit crystalline iron terephthalate coordination network thin films from FeCl_3_ and TPA precursors. The process yields highly crystalline films with the rate of ca. 11 Å/cycle in the temperature range from 240 to 260 °C. The trivalent state of iron was confirmed by XPS, and the bonding and crystal structures were discussed based on FTIR and GIXRD data. The former results confirmed that iron in the films is bonded to the organic moiety through both of its carboxylate oxygen atoms while the latter results somewhat pointed towards a MOF-2 type crystal structure; the detailed crystal structure however remained to be clarified in future studies. Depositions using the same precursors at temperatures below 240 °C yielded amorphous films. Amorphous Fe-TP films resulted also from the depositions based on the Fe(acac)_3_ precursor, in this case within the entire deposition temperature range investigated. Recalling also our previous work on Co-TP and Mn-TP depositions yielding amorphous films from both acac and thd metal precursors^21^, we conclude that the steric hindrance caused by the bulky ligands apparently complicates the building-up of the new coordination bonds and thereby the coordination network structure. This provides important guidelines for the *in-situ* gas-phase deposition of transition-metal based coordination network thin films – a target of great future demand.

## Methods

### Samples

In the ALD/MLD growth of our Fe-TP films, the inorganic and organic precursors were sequentially pulsed into a commercial flow-type hot-wall ALD reactor (F-120 by ASM Microchemistry Ltd) separated with N_2_ purge pulses (>99.999%; Schmidlin UHPN 3000 generator). Silicon(100) wafers cut into 3 × 3 cm^2^ pieces were used as substrates. For most of the depositions FeCl_3_ (Merck, 95%) was employed as the inorganic precursor, but we also carried out experiments with Fe(acac)_3_ (Aldrich, 99.9%) for comparison. In both cases terephthalic acid (Tokyo Chemical Industry CO., Ltd, >99.0%) was used as the organic precursor. The solid precursors were evaporated from open precursor boats placed inside the ALD reactor at the temperatures of 155, 120 and 185 °C, respectively. Different precursor pulse lengths were initially tested at different deposition temperatures for the precursors, FeCl_3_ and TPA, in the ranges of 0.5–8 s and 5–35 s, respectively. For most of the further depositions the precursor pulse/N_2_ purge lengths were fixed to 4 s/8 s for FeCl_3_ and 20 s/40 s for TPA. The pressure was 2–4 mbar and the N_2_ flow 300 sccm during the depositions.

### Characterization

Grazing incidence X-ray diffraction (GIXRD; X’Pert MPD PRO Alfa 1, PANalytical; Cu Kα radiation) was used to determine the crystallinity of the Fe-TP thin films. The same equipment was used to measure the film thickness in X-ray reflectivity (XRR) setup. All though the samples were relatively stable in the air the thickness measurements for the as-deposited films were carried out within 15 min after taking the samples out from the reactor to get as reliable results as possible. The data were fitted by X’Pert Reflectivity software by PANalytical which additionally yielded the roughness values for the samples.

Fourier transform infrared (FTIR; Nicolet magma 750) spectroscopy and X-ray photoelectron spectroscopy (XPS; Axis Ultra, Kratos Analytical, Manchester, UK) were used to determine the chemical structure of the thin films. In FTIR dry air was used for purging the chamber during the measurements. A spectrum of blank Si was subtracted from the spectra to compensate the interference caused by the substrate. In XPS the spectra for each element were normalized with the main peak of the carbon. Data sets for each sample were collected from the analysis area of less than 1 mm^2^ with depth less than 10 nm. Elemental surface concentrations were determined from the wide-energy scans, while the high-resolution regions were used for compound identification (resolution PE 20 eV, step 0.1 eV). CasaXPS was used as the analysis software.
